# Neuropsychiatric Manifestations in Methylmalonic Acidemia and Homocystinuria of Adult Presentation. A Case Report

**DOI:** 10.1192/j.eurpsy.2026.12142

**Published:** 2026-07-31

**Authors:** P. E. Paredes, C. B. Fresno, H. B. Darriba

**Affiliations:** 1 Psiquiatria, Hospital Reina Sofia, Tudela; 2 Psiquiatria, Hospital Universitario Miguel Servet, Zaragoza, Spain

## Abstract

**Introduction:**

Each neurometabolic disorder is individually rare, but their cumulative incidence is relatively high, around 1/1,500 to 1/5,000 live births.

In neurometabolic diseases, there is a deficit in the transformation of a substrate that prevents the production of one or more enzymes.

Common neuropsychiatric manifestations associated with vitamin B12 deficiency include motor, sensory, and autonomic symptoms, cognitive impairment, mood disorders, and psychotic symptoms.

Psychiatric manifestations are considered a particular form of presentation which, precisely because of their isolated nature, make diagnostic identification difficult, given that behavioral changes in these NME generally have an insidious onset.

**Objectives:**

Objective: To report the neuropsychiatric alterations associated with inborn errors of vitamin B12 metabolism in adults

**Methods:**

Case report.

**Results:**

30-year-old woman who starts with anxiety, difficulty in daily organization, suspected to be reactive to active mourning and initiation of Duncan’s hyperproteic diet; 7 months later she debuts with acute encephalopathy (confusion, bradypsychia, parkinsonism), admitted to ICU for focal status epilepticus coinciding with the initiation of parenteral feeding. Brain MRI showed pericerebellar and basal ganglia hyperintensity, although LP showed no alterations, Hashimoto’s encephalopathy was initially diagnosed with minimal response to corticosteroids. The evolution in the following 4 months was torpid, persisting frontal-subcortical dysfunction, with bradypsychia, loss of skills, memory, language, gait and behavioral alterations (irritability, agitation), as well as psychotic symptoms with polymorphous delusional ideation: of harm, control and pregnancy with auditory hallucinations. She was admitted to Neurology to study the picture and was referred to Psychiatry. Due to hyperhomocysteinemia and hyperammonemia and aciduria in urine, genetic screening was performed to rule out alteration of Vitb12 metabolism, diagnosing methylmalonic aciduria with homocystinuria due to deficiency in MMACHC (cbIC) (anomaly in the synthesis of adenosylcobalamin (AdoCbl) and methylcobalamin (MeCbl), which are coenzymes derived from Vitb12). She was treated with Vit B12 and B9 supplements, L-carnitine, betaine, and paliperidone, with significant early improvements in biochemical parameters, and later in clinical manifestations. After discharge he did not present psychosis again.

**Conclusions:**

alterations in cobalamin metabolism may present psychiatric alterations. The manifestations are usually very polimorfic.

**Disclosure of Interest:**

None Declared

